# Renalase gene Glu37Asp polymorphism affects susceptibility to diabetic retinopathy in type 2 diabetes mellitus

**DOI:** 10.1007/s00592-021-01740-8

**Published:** 2021-06-22

**Authors:** Monika Buraczynska, Karolina Gwiazda-Tyndel, Bartłomiej Drop, Wojciech Zaluska

**Affiliations:** 1 Department of Nephrology, Medical University of Lublin, Jaczewskiego 8, 20-950 Lublin, Poland; 2 Department of Medical Informatics and Statistics, Medical University of Lublin, Lublin, Poland

**Keywords:** Renalase gene, Type 2 diabetes, Diabetic complications, Polymorphism, Risk allele

## Abstract

**Aims:**

Renalase (RNLS) is an enzyme with monoamine oxidase activity that metabolizes circulating catecholamines. The RNLS gene Asp37Glu missense polymorphism (rs2296545) has been associated with hypertension, cardiac hypertrophy and dysfunction, and stroke. The purpose of our study was to investigate the potential involvement of this polymorphism in the microvascular complications of type 2 diabetes (T2DM).

**Methods:**

In this case–control study, the polymorphism was genotyped in 860 patients with T2DM and 400 healthy controls. The genotype and allele distribution was compared in subgroups of patients: with diabetic nephropathy (DN+) (*n* = 405) versus DN− (independently of the presence of DR) and, similarly, patients with diabetic retinopathy (DR+) (*n* = 328) versus DR− (independently of the presence of DN).

**Results:**

No significant association was detected between analyzed polymorphism and DN. In contrast, the retinopathy subgroup showed a significantly higher frequency of G allele (OR 1.4, 95% CI 1.16–1.72, *p* = 0.0005) and GG genotype (OR 1.86, 95% CI 1.26–2.75, *p* = 0.001) than DR− patients. The effect of RNLS Glu37Asp polymorphism on DR remained significant after adjustments for age, gender, BMI, and duration of T2DM (*p* = 0.005).

**Conclusions:**

This is the first study to investigate RNLS gene polymorphism in microvascular complications of T2DM. The results suggest that RNLS rs2296545 SNP might be considered a risk factor for diabetic retinopathy in T2DM patients. This can provide new insight into the role of renalase gene in the pathophysiology of microvascular complications of diabetes.

## Introduction

Type 2 diabetes mellitus (T2DM), a heterogeneous disorder of glucose metabolism, accounts for close to 90% of all diabetes cases, contributing to the burden on individuals and the health care system (American Diabetes Association 2014) [1]. Despite the availability of effective pharmacological treatments, glycemic control deteriorates and persistent hyperglycemia results in microvascular complications, such as diabetic nephropathy, retinopathy, and neuropathy. Diabetic microvascular complications are the main cause of morbidity and premature mortality in patients with T2DM [2–4]. Multiple genetic and environmental factors contribute to the pathogenesis of type 2 diabetes and its complications [5]. Micro- and macrovascular complications of diabetes share some underlying pathophysiology and risk factors. Hypertension, a common comorbidity in individuals with type 2 diabetes, further contributes to macro- and microvascular complications. In this regard, renalase gene polymorphisms demonstrated a significant role in hypertension and cardiovascular complications development [6].

Renalase represents a new class of flavin-adenine dinucleotide (FAD) containing monoamine oxidases. It is expressed mainly in kidney and heart and metabolizes circulating catecholamines [7, 8]. Decreased plasma levels of renalase contribute to hypertension due to the reduced degradation of catecholamine [9]. Renalase has significant hemodynamic effects and is involved in the regulation of blood pressure and cardiovascular function [10–15].

Human renalase is encoded by the gene (RNLS) localized on chromosome 10 (10q23.33). It has 10 exons covering 311 000 bp [10]. Numerous single nucleotide polymorphisms (SNPs) were identified in the renalase gene, with those most studied localized at the potential functional domains. In the previous studies, several SNPs were reported to be associated with coronary heart disease [16, 17], stroke [18], hypertension [6, 19], and end-stage renal disease [20].

To date, most of the analyses focused on a common missense polymorphism resulting in the amino acid change (glutamic acid to aspartic acid, Glu37Asp). This polymorphism was associated with hypertension, cardiac hypertrophy and dysfunction, and ischemia [21]. Other studies also reported its association with cardiovascular disease and hypertension [6, 17, 22].

The association between renalase gene polymorphisms and microvascular complications of diabetes has not been previously investigated. Thus, the purpose of the present study was to assess the potential involvement of renalase gene rs2296545 polymorphism in diabetic nephropathy and retinopathy in type 2 diabetes patients.

## Methodology

### Patients and controls

Our study comprised 860 unrelated patients with type 2 diabetes lasting > 10 years (mean age 59.4 ± 9.3 years). All enrolled subjects were Caucasian of Polish origin. Before inclusion into the study, a written informed consent was provided by all patients and healthy controls in accordance with standards of the 1964 Declaration of Helsinki. The detailed protocol of the study was approved by the institutional ethics committee.

Diabetes was diagnosed on the basis of American Diabetes Association criteria [1]. One or more of the following conditions were required for diagnosis: the classic symptoms of hyperglycemia (polyuria, polydipsia, loss of weight), fasting plasma glucose level > 7 mmol/l or random level > 11 mmol/l, and the use of insulin or oral hypoglycemic medication. All subjects underwent complete physical examination that included fasting plasma glucose, glycated hemoglobin (HbA1c), full lipid profile, albumin-to-creatinine ratio (ACR), albumin excretion rate (AER), and body mass index (BMI). This was a cross-sectional study, so the patients were not matched for demographic or medical characteristics.

Upon enrollment in the study, all patients were evaluated for renal function and ophthalmological status. Their medical records were reviewed, and additional tests were performed where needed and diagnoses verified.

For analyzing the effect of renalase gene polymorphism, patients were assigned to three subgroups:

- *DN* patients with diabetic nephropathy (with or without retinopathy);
- *DR* patients with diabetic retinopathy (with or without nephropathy);
- *No MVC* patients with no microvascular complications.

A total of 405 patients presented with diabetic nephropathy, of whom 194 patients (47.9%) demonstrated concomitant diabetic retinopathy of any stage. Diabetic nephropathy was diagnosed in the presence of persistent albuminuria ≥ 300 mg/24 h in two consecutive measurements performed 1 month apart, in the absence of hematuria or infection. The presence of urinary tract infection, other renal diseases, neoplastic, rheumatological or endocrine diseases, and significant heart failure were the exclusion criteria. Patients with microalbuminuria were excluded.

Diabetic retinopathy was initially diagnosed by independent ophthalmologists in 328 patients after an eye examination including visual acuity, fundoscopic examination, and fundus photography. After enrollment in the study, findings from fundoscopic examination were assessed and verified by a retinal specialist. Retinopathy was diagnosed according to the Early Treatment Diabetic Retinopathy Study (ETDRS) criteria [23]. Exclusion criteria for the DR subgroup consisted of poor image quality, any dioptric media opacities, concomitant ocular disorders, particularly retinal vascular disorders, maculopathies, or optic nerve disorders. Patients who received recent retinal photocoagulation or intraocular surgery were also excluded.

Out of the type 2 diabetes patients, 194 had both complications, diabetic nephropathy and diabetic retinopathy.

We also included a group of 321 T2DM patients in no MVC group (mean age 58.3 ± 9.2 years). The clinical evaluation and complete medical documentation confirmed the lack of complications in these patients.

The control group (*n* = 400, mean age 57.5 ± 8.1 years) consisted of unrelated healthy volunteers (mostly blood donors and hospital staff members) who underwent health examination. They had no known history of diabetes, cardiovascular or renal disease. The subjects with the family history of these diseases in first-degree relatives were excluded. This group was first genotyped to estimate genotype/allele frequencies.

### Determination of rs2296545 genotype

Genomic DNA was extracted from peripheral blood leukocytes (stored at − 70 °C) obtained by the standard procedure [24]. The RNLS SNP rs2296545 was analyzed by amplification of 209 bp DNA fragment by polymerase chain reaction (PCR). The following specific primer pair was used: forward 5ʹ-GGAAGTCCCCGATCACGT GAC-3ʹ and reverse 5ʹ-TGCTGTGTGGGACAAGGCTGA-3ʹ. Template DNA (200 ng) was amplified in a 30 μl reaction. The predenaturation step was at 95 °C for 5 min and was followed by 35 cycles consisting of denaturation at 95 °C, annealing at 60 °C, and, extension at 72 °C (1 min each). The final extension step was at 72 °C for 7 min. After PCR, the amplicon (10 μl) was digested with 5 units of Eco81 I restriction endonuclease (Thermo Fisher Scientific) (37 °C for 12 h). The resulting DNA fragments were analyzed by electrophoresis in 2.5% agarose gel. The length of products was 188 bp + 21 bp for the C allele and 209 bp for the G allele. The quality of genotyping was controlled by using blind DNA duplicates (96 samples). The rate of concordance was 100%. Also, 20 random samples for each genotype were sequenced with a forward primer by automated sequencing in the CEQ 8000 Genetic Analysis System (Beckman Coulter).

### Statistical analysis

Statistical calculations were performed using SPSS 18.0 for Windows (SPSS, Inc., Chicago, IL). For comparison of baseline characteristics, the normally distributed continuous variables are presented as means ± SD. The Hardy–Weinberg equilibrium was tested using the chi-square test. Genotype distribution and allele frequencies were compared between groups and subgroups using a chi-square test of independence with 2 × 2 contingency and *z* statistics. Mann–Whitney test and Pearson’s *Χ*^2^ test of independence were used for comparing continuous and categorical variables. Where appropriate, the odds ratios (OR) with corresponding 95% confidence intervals (CI) were calculated for associations. An interaction of the studied polymorphism with the risk factors for diabetes and its complications was assessed with multivariate logistic regression analysis. A two-tailed type I error rate of 5% was considered statistically significant. Post hoc power calculations were done using the available on-line power calculator.

## Results

### Demographic and clinical characteristics

The genotype of the rs2296545 SNP in the renalase gene was determined in 860 patients with type 2 diabetes and 400 healthy controls with genotyping success rate 100%. The demographic and clinical characteristics of subjects included in the study are presented in Table 1. Among the patients with T2DM, 405 (47%) had diabetic nephropathy, of whom 194 patients (47.9%) had concomitant diabetic retinopathy.

**Table 1:** Clinical characteristics of studied subjects

Variables	T2DM patients	DN	DR	Without MVC	Controls
N	860	405	328	321	400
Male/female ratio	462/398	208/197	175/153	161/160	205/195
Age at study (years)	59.4 ± 9.3	59.2 ± 10	58.8 ± 9.4	58.3 ± 9.2	57.5 ± 8.1^a^
Age at diabetes diagnosis (years)	45.2 ± 7.3	44.3 ± 8.3	43.8 ± 8.6	46 ± 1.3^b^	NA
Diabetes duration (years)	14.9 ± 7.3	14.5 ± 8.6	15.4 ± 8.1	14.0 ± 7.2	NA
Cardiovascular disease (%)	669 (77.8)	318 (78.6)	260 (79.3)	209 (65)^b^	0^a^
Hypertension (%)	628 (73)	318 (78.5	246 (75)	218 (68)	0^a^
Diabetic nephropathy (%)	405 (47.1)	405 (100)	194 (59)^c^	0	0^a^
Diabetic retinopathy (%)	328 (38.1)	194 (48)	328 (100)^c^	0	0^a^
Total cholesterol (mmol/l)	4.9 ± 0.8	4.86 ± 1.0	4.92 ± 0.9	4.6 ± 0.76^b^	4.0 ± 0.78^a^
Triglyceride (mmol/l)	2.3 ± 1.26	2.3 ± 0.96	2.1 ± 1.42^c^	1.9 ± 0.83^b^	1.2 ± 0.63^a^
HbA_1c_ (%)	8.1 ± 2.5	8.3 ± 2.7	8.2 ± 3.3	8.3 ± 2.6	ND
BMI (kg/m^2^)	28.4 ± 8.6	28.2 ± 5.6	28.2 ± 9.3	27.3 ± 4.2	27.1 ± 4.2^a^

*T2DM* type 2 diabetes; *DN* diabetic nephropathy (either DR+ or DR−); *DR* diabetic retinopathy (either DN+ or DN−); *MVC* microvascular complications; *BMI* body mass index; *HbA*_*1c*_ glycated hemoglobin; *NA* not applicable; *ND* not determined. For continuous characteristics values are presented as means ± SD. For discrete characteristics values are numbers and percentage (in parentheses). ^a^*p* < 0.05 vs. T2DM, ^b^*p* < 0.05 Versus. DN and DR, ^c^*p* < 0.05 Versus DN. Groups compared by Mann–Whitney and Pearson’s *Χ*^2^ tests

Diabetic retinopathy was diagnosed in 328 patients (38%), of whom 194 patients (59%) had concomitant diabetic nephropathy. The gender distribution was similar in T2DM patients and control group but there was a statistically significant difference in the age at study. There was also a difference in the lipid profile and BMI between these groups. The clinical characteristics were similar between patients with diabetic nephropathy and those with diabetic retinopathy, except for triglyceride levels.

### Association of rs2296545 with T2DM

Table 2 compares the distribution of the studied polymorphism in T2DM patients and healthy individuals. The frequency of genotypes for rs2296545 SNP in the control group did not deviate from the Hardy–Weinberg equilibrium (*p* = 0.38), suggesting that the subjects are representative for the population. There was a slight deviation in T2DM group (*p* = 0.034). The genotype and allele distribution did not differ significantly between the T2DM patient group and control individuals.

**Table 2:** Genotype and allele distribution of Glu37Asp polymorphism in the RNLS gene in T2DM patients and controls

	Genotypes				MAF	OR (95% CI)	
	N	CC	CG	GG		G allele	GG genotype^a^
T2DM patients	860	276 (32)	395 (46)	189 (22)	0.45	0.92 (0.8–1.1)	0.88 (0.6–1.2)
						*p* = 0.33	*p* = 0.46
Controls	400	108 (27)	208 (52)	84 (21)	0.47	Ref	Ref

*T2DM* type 2 diabetes mellitus; *RNLS* renalase; *MAF* minor allele frequency. Genotype distribution is shown as numbers (%). Hardy–Weinberg equilibrium: *χ*^2^ = 0.765, *p* = 0.381 for control group; *χ*^2^ = 4.445, *p* = 0.034 for T2DM patients. ^a^Calculated versus CC genotype

### Association of rs2296545 SNP with microvascular complications

Genotype distribution and allele frequency were compared in the subgroups of T2DM patients with diabetic complications and those without complications. To analyze the association of the Glu37Asp polymorphism and the risk of diabetic nephropathy, we compared the genotype and allele distribution between DN+ patients (*n* = 405) and DN− patients (*n* = 455). For the risk of diabetic retinopathy, DR+ patients (*n* = 328) were compared with DR− subgroup (*n* = 532). The detailed results are presented in Table 3. There was no statistically significant association detected between analyzed polymorphism and diabetic nephropathy. In contrast, the DR+ subgroup showed a significantly higher frequency of the G allele (OR 1.4, 95% CI 1.16–1.72, *p* = 0.0005) and GG genotype (OR 1.86, 95% CI 1.26–2.75, *p* = 0.001) than DR− patients. Also, when compared with no MVC subgroup, DR+ patients had higher frequency of the risk allele (OR 1.81, 95% CI 1.17–2.8, *p* = 0.0078). Since more than half of the DR+ patients had concomitant DN, we further analyzed the distribution of Glu37Asp polymorphism in DR+ patients according to the presence or absence of DN (Table 4). The polymorphism distribution did not differ significantly between T2DM DR+ patients with and without concomitant DN.

**Table 3:** Genotype and allele distribution of Glu37Asp polymorphism in the RNLS gene in subgroups of T2DM patients

	Genotypes				MAF	OR (95% CI)	
	N	CC	CG	GG		G allele	GG genotype^a^
T2DM DN+	405	114 (28)	198 (49)	93 (23)	0.47	1.2 (0.99–1.46)	1.37 (0.94–1.99)
						*p* = 0.052	*p* = 0.092
T2DM DN−	455	162 (36)	197 (43)	96 (21)	0.43	Ref	Ref
T2DM DR+	328	78 (24)	170 (52)	80 (24)	0.50	1.41 (1.16–1.72)	1.86 (1.26–2.75)
						*p* = 0.0005	*p* = 0.001
T2DM DR−	532	198 (38)	225 (42)	109 (20)	0.42	Ref	Ref
T2DM without MVC	321	113 (35)	144 (45)	64 (20)	0.42	1.37 (1.1–1.71)^b^	1.81 (1.17–2.8)^b^
						*p* = 0.0042	*p* = 0.0078

*T2DM* type 2 diabetes mellitus; *RNLS* renalase; *MAF* minor allele frequency; *DN* diabetic nephropathy (either DR+ or DR−); *DR* diabetic retinopathy (either DN+ or DN−); *MVC* microvascular complications. Genotype distribution is shown as numbers (%). ^a^Calculated versus CC genotype. ^b^Calculated versus T2DM with DR subgroup

**Table 4:** Genotype and allele distribution of Glu37Asp polymorphism in the RNLS gene in the T2DM DR+ patients according to the presence or absence of DN

	Genotypes				MAF	OR (95% CI)	
	N	CC	CG	GG		G allele	GG genotype^a^
T2DM DR+ DN−	134	35 (26)	66 (49)	33 (25)	0.49	1.38 (1.03–1.84)	1.66 (0.94–2.93)
						*p* = 0.028	*p* = 0.047
T2DM DR+ DN+	194	43 (22)	104 (54)	47 (24)	0.51	1.42 (1.1–1.82)	1.76 (1.06–2.93)
						*p* = 0.006	*p* = 0.028
T2DM without MVC	321	113 (35)	144 (45)	64 (20)	0.42	ref	ref

*T2DM* type 2 diabetes mellitus; *RNLS* renalase; *MAF* minor allele frequency; *DN* diabetic nephropathy; *DR* diabetic retinopathy; *MVC* microvascular complications. Genotype distribution is shown as numbers (%). ^a^Calculated versus CC genotype. OR calculated for DR+ DN− versus DR+ DN+ was 0.93 (0.68–1.27), *p* = 0.654 and 0.88 (0.43–1.62), *p* = 0.645, for the G allele and GG genotype, respectively

Multiple logistic regression analysis showed that only the *RNLS* gene polymorphism was a significant predictor of diabetic retinopathy. The effect of RNLS Glu37Asp polymorphism on diabetic retinopathy remained significant even after adjustments for age, gender, BMI, and duration of T2DM (*p* = 0.005) (Table 5). Table 6 shows comparison of clinical characteristics between patients with and without diabetic retinopathy. Patients in DR− subgroup were older than those in DR+ subgroup. The proportion of patients who also had nephropathy was significantly lower in DR− than in the DR+ subgroup. Plasma lipids and other metabolic parameters showed no difference between the subgroups except for triglyceride levels that were higher in DR− patients.

**Table 5:** Multivariate logistic regression analysis

Variable	Odds ratio	95% CI	*p* value
Age at study	1.16	0.73–1.41	0.07
Gender	1.28	0.82–1.67	0.09
T2DM duration	1.19	0.72–1.83	0.13
BMI	1.12	0.92–1.21	0.24
G allele*	1.37	1.19–2.06	0.005

*T2DM* type 2 diabetes mellitus. *RNLS Glu37Asp polymorphism

An unconditional model of multiple logistic regression analysis was used for interaction of *RNLS* gene polymorphism with other variables

**Table 6:** Clinical characteristics of T2DM patients with and without retinopathy

Variables	DR+	DR−	*P* value
N	328	532	
Male/female ratio	175/153	287/245	0.886
Age at study (years)	58.8 ± 9.4	60 ± 9.2	0.065
Age at diabetes diagnosis (years)	43.8 ± 8.6	46.7 ± 5.9	0.0001
Diabetes duration (years)	15.4 ± 8.1	14.4 ± 6.5	0.046
Cardiovascular disease (%)	260 (79.3)	409 (76.8)	0.302
Diabetic nephropathy (%)	194 (59)	211 (39.6)	< 0.0001
Total cholesterol (mmol/l)	4.92 ± 0.9	4.88 ± 0.8	0.497
Triglyceride (mmol/l)	2.1 ± 1.42	2.5 ± 1.2	< 0.0001
HbA_1c_ (%)	8.2 ± 3.3	8.0 ± 1.6	0.234
BMI (kg/m^2^)	28.2 ± 9.3	28.6 ± 7.9	0.5

*T2DM* type 2 diabetes; *DR* diabetic retinopathy (either DN+ or DN−). For continuous characteristics values are presented as means ± SD. For discrete characteristics values are numbers and percentage (in parentheses)

Among 328 patients with diabetic retinopathy, 134 (41%) had proliferative retinopathy (PDR). When compared between NPDR and PDR patients, there were no significant differences in the Glu37Asp allele and genotype distribution between these subgroups (*p* > 0.05).

## Discussion

The macrovascular and microvascular complications of diabetes share some pathophysiology and conventional as well as genetic risk factors. The previous studies demonstrated that renalase gene polymorphisms are involved in the macrovascular complications of diabetes, such as hypertension and cardiovascular disease [6, 16, 17, 21, 25, 26]. Therefore, in the present case–control study, we aimed to assess the potential involvement of the RNLS gene rs2296545 polymorphism in microvascular complications of type 2 diabetes mellitus.

It was previously documented that renalase is involved in diabetes and its clinical features. The levels of renalase in the T1DM and T2DM groups of patients were significantly higher than in control group and were correlated with changes in blood pressure, glomerular filtration, and insulin resistance [15]. In our study no statistically significant differences were observed when the distribution of polymorphism was compared between T2DM patients and healthy controls. This replicates the result from our previous study, where rs2296545 polymorphism was associated with hypertension in type 2 diabetes patients but not with diabetes itself [18]. At present, there are no other molecular studies published on the effects of the renalase gene polymorphisms in type 2 diabetes. There are some reports from linkage and association studies, linking the renalase gene with type 1 diabetes, and suggesting an immune basis for the disease. In a genome-wide association study, the human leukocyte antigen (HLA) region, affecting the risk of T1DM, contained the rs10509540 SNP in the renalase gene [27]. Another study of T1DM-associated regions confirmed a potential role of renalase in the development of autoimmune pancreatic β-cell destruction [28]. The rs10509540 polymorphism has been shown to have, in interaction with IL-2 gene polymorphism, an age-at-diagnosis effect on pediatric-onset T1DM [29]. The association was also found between RNLS rs10887800 polymorphism and gestational diabetes mellitus [30].

We further assessed the polymorphism distribution in the subgroups of T2DM patients with diabetic nephropathy and retinopathy. While there was no association detected between this SNP and diabetic nephropathy, the retinopathy subgroup showed a significant association of the G allele with DR. This association remained significant after adjustment for age, gender, BMI, and duration of T2DM. The polymorphism distribution did not differ significantly between T2DM DR+ patients with and without concomitant DN. These findings demonstrate that the renalase gene polymorphism is associated with susceptibility to retinopathy in type 2 diabetes. The reason for the discrepancy in the effect of rs2296545 polymorphism between DR and DN is unclear at this point. It might be related to heterogeneity in the genetic susceptibility due to different pathophysiological pathways in the development of these microvascular complications. It is also possible that the effect size on the renal outcome might be much smaller than on ocular outcome. The lack of association of the studied polymorphism with diabetic nephropathy is difficult to explain in the light of the links between renalase and kidney disease widely described in the literature. Concentrations of renalase in serum are significantly higher in patients with chronic kidney disease (CKD) than in control group and are elevated in the increasing stage of renal failure [31–33]. Zbroch et al. described a significant inverse correlation between the serum renalase and residual renal function [34]. In an elegant study on the role of renalase in diabetic renal injury, using a mouse model of renalase knockout mice, the authors showed the protective role of renalase in DN. The heterozygous knockout of renalase in db/db mouse caused an exacerbation of albuminuria, mesangial expansion, and renal injury progression. These symptoms were ameliorated with renalase overexpression in db/db mice. Renalase exerted the protective effects in DN through inhibition of mesangial cell hypertrophy, oxidative stress, and inflammation [35]. The results of the molecular studies also showed a linkage between the renalase gene polymorphisms and kidney diseases [36, 37].

The exact mechanism of association between Glu37Asp polymorphism in the renalase gene and the risk of developing DR is not clear yet and requires further, larger studies, also on the cellular level. The mechanisms that initiate and control the progress of diabetic retinopathy are still unclear. It is hard to explain why not all patients after a long diabetes duration, even with poor glycemia, develop DR. On the other hand, some patients with good glycemic control develop a vision-threatening DR. Our finding could be interpreted as an effect of the rs2296545 polymorphism on certain clinical or metabolic factors associated with retinopathy. Endothelial dysfunction plays a crucial role in the early pathophysiology of vascular complications of diabetes [38]. Yun et al. identified endothelial dysfunction as an independent predictor of an increased DR prevalence in patients with T2DM [39]. The progressive dysfunction of endothelial cells contributes to the retinal morphological and pathophysiological changes: thickening of capillary basement membrane, perivascular cell loss, damage of blood-retina barrier, and neovascularization [40, 41]. An important feature of endothelial dysfunction is an increased production and biological activity of the potent vasoconstrictor and proinflammatory peptide endothelin-1 (ET-1). Several lines of evidence suggest that the ET-1 system contributes to diabetic vascular disorders. Elevated renalase levels increase serum ET-1 levels, thus aggravating endothelial dysfunction and vasoconstriction [33]. Vascular and extravascular sites in the retina are a rich source of ET-1 expression. The ET-1 impairs the autoregulation of retinal blood flow, which can cause hyperperfusion, promoting formation of retinal microaneurysms and edema [42]. On the other hand, ET-1-mediated vasoconstriction can trigger a hypoxic state which later leads to pathological angiogenesis as seen in diabetic retinopathy [43]. The relationship between ET-1 plasma concentration and a degree of progression of DR has been demonstrated in many studies [44]. Although the role of renalase in endothelial function might be an important factor in developing DR, this mechanism is still unclear and requires further biological and functional studies.

The results obtained in our study should be interpreted with caution. The rs2296545 SNP is located in exon 2 of the RNLS gene and, therefore, it might change the gene functions via affecting the protein structure or function. However, to date, there have been no results from functional studies published to support this speculation. The important limitation of our study is that we did not also analyze the functional consequences of this polymorphism. The determinations of serum renalase levels or its enzymatic activity were not performed in the study subjects.

In conclusion, to the best of our knowledge, this is the first published study investigating the effect of the RNLS gene polymorphism on microvascular complications of diabetes. Our findings demonstrate that the rs2296545 polymorphism in the renalase gene is associated with increased susceptibility to retinopathy in type 2 diabetes patients. A novel observation of this association as well as its clinical significance needs to be confirmed in further, larger studies.
